# Pulsed administration for physiological estrogen replacement in mice

**DOI:** 10.12688/f1000research.54501.1

**Published:** 2021-08-16

**Authors:** Carmen Corciulo, Julia M. Scheffler, Karin L. Gustafsson, Christina Drevinge, Piotr Humeniuk, Alicia M. del Carpio Pons, Matti Poutanen, Claes Ohlsson, Marie K. Lagerquist, Ulrika Islander

**Affiliations:** 1 Sahlgrenska Osteoporosis Centre, Centre for Bone and Arthritis Research, Department of Internal Medicine and Clinical Nutrition, Sahlgrenska Academy at the University of Gothenburg, Gothenburg, 413 45, Sweden; 2 Institute of Biomedicine, Research Centre for Integrative Physiology and Pharmacology, Turku Center for Disease Modeling, University of Turku, Turku, Kiinamyllynkatu 10, FI-20520, Finland; 3 Department of Drug Treatment, Sahlgrenska University Hospital, Gothenburg, 41345, Sweden

**Keywords:** Estrogens, sex steroids, therapy, ovariectomy, osteoporosis

## Abstract

Estrogens are important regulators of body physiology and have major effects on metabolism, bone, the immune- and central nervous systems. The specific mechanisms underlying the effects of estrogens on various cells, tissues and organs are unclear and mouse models constitute a powerful experimental tool to define the physiological and pathological properties of estrogens. Menopause can be mimicked in animal models by surgical removal of the ovaries and replacement therapy with 17β-estradiol in ovariectomized (OVX) mice is a common technique used to determine specific effects of the hormone. However, these studies are complicated by the non-monotonic dose-response of estradiol, when given as therapy. Increased knowledge of how to distribute estradiol in terms of solvent, dose, and administration frequency, is required in order to accurately mimic physiological conditions in studies where estradiol treatment is performed. In this study, mice were OVX and treated with physiological doses of 17β-estradiol-3-benzoate (E2) dissolved in miglyol or PBS. Subcutaneous injections were performed every 4 days to resemble the estrus cycle in mice. Results show that OVX induces an osteoporotic phenotype, fat accumulation and impairment of the locomotor ability, as expected. Pulsed administration of physiological doses of E2 dissolved in miglyol rescues the phenotypes induced by OVX. However, when E2 is dissolved in PBS the effects are less pronounced, possibly due to rapid wash out of the steroid.

## Introduction

Steroid hormones control the physiology of the whole body. Alterations of this well-balanced system can lead to organ dysfunction, representing a risk factor for many human diseases ^1^. Women are more subjected to changes in sex-hormone concentrations compared to men due to the menstrual cycle and they also go through menopause, which is defined by lack of the menstrual cycle for more than 12 consecutive months. This phase of women's lives is characterized by reproductive senescence and a decrease of sex-hormone production ^2^. Many medical conditions are associated with menopause, including cardiovascular diseases (affecting 75% of postmenopausal women) ^3^, hot flushes (85%) ^4^, vaginal dryness and genitourinary syndrome (75%) ^5– 8^, bone loss and consequent osteoporosis (30%) ^9, 10^, emotional symptoms leading to sleep disturbance (50%) ^11^, irritability (42%) and depressive mood (29%) ^12, 13^. The association between menopause and these conditions emphasizes the importance of female sex-hormones (estrogens among them) in maintaining women's health. Thus, much research worldwide is focused on determining the effects of estrogens on *e.g.,* bone turnover, behavior and pain sensitivity, cardiac alterations, and influence on the immune system.

Estrogens are expressed in all vertebrate species, as well as in some of the invertebrates. They are produced by the ovaries and are considered the primary female sex hormones ^14^. Natural estrogens comprise a class of compounds including estrone, estradiol (17α-estradiol and 17β-estradiol), and estriol. 17β-estradiol is the most abundant and biologically active form of estrogen produced in non-pregnant premenopausal women ^15^.

Animal models that mimic human reproductive senescence, including mouse models, are powerful scientific tools to study mechanisms mediated by estrogens-, to discover new and safer hormone-based drugs, and to predict the outcome of therapeutic treatments. Various approaches for estrogen replacement therapy in mice have been described in the literature, all displaying different strengths and weaknesses. In addition, various doses and patterns of administration have been used with distinctive outcomes ^16– 18^. The design of an experiment that includes treatment with estrogens is complicated by the difficulty to predict the effect of the hormones at higher doses based on data at lower doses. Indeed, estrogens have different affinity for the estrogen receptors, ERα and ERβ, and the expression and function of the receptors are regulated depending on the dose of the ligand. This mechanism of mutual regulation results, in some tissues, in a non-monotonic dose-response, meaning that increasing doses of hormones generates dissimilar or opposite effects compared with lower doses, giving a U-shaped dose-response curve ^19^.

The most common methods for estradiol delivery in mice are subcutaneous implants of estradiol pellets or silastic tubes filled with estradiol powder. Neither of these procedures guarantee the release of a controlled amount of estradiol, as a high amount of hormone is delivered during the days directly after the implant, and this peak is subsequently followed by a drastic reduction ^17, 20^. Chow supplemented with estrogens has been largely described as a convenient method of administration with low stress for the animals but with uncertainty in the dose delivered ^18^. Implants of an osmotic mini-pump would overcome this problem since the pores in the filters regulate the amount of solution released. Nevertheless, the size of the osmotic pump and consequently the volume of the solution loaded is limited by the small size of the animals. Moreover, the surgical removal of the pump is needed after 4 weeks, making this delivery method un-suitable for longer experiments. Most importantly, for all these techniques, the constant hormone release does not mirror the normal cyclic fluctuations of estradiol in females.

Another usual method for estradiol delivery to mice is repeated injections. For this technique, the vehicle used to dissolve estradiol needs to be considered. In common practice, estradiol is dissolved in an oil-based vehicle and injected subcutaneously. The oil is slowly absorbed, creating pockets of oil that persist for many days. In this method of administration, it is likely that the injected hormone is not completely absorbed at the time of the next injection, leading to estradiol accumulation and uneven release over time.

The present study aimed to determine whether pulsed subcutaneous injections with 17β-estradiol-3-benzoate (E2) every 4 days in healthy ovariectomized (OVX) mice can be used to better mimic the physiological dose of endogenously produced E2. Two different preparations of E2 were tested, either suspended in miglyol or in phosphate buffer saline (PBS), in order to determine the optimal solvent for injections of E2. We report the effects of pulsed E2 administration in OVX mice on sex steroid concentration in serum, soft tissues, bone, and motor ability of the mice.

## Methods

### Animals

**Figure 1.**
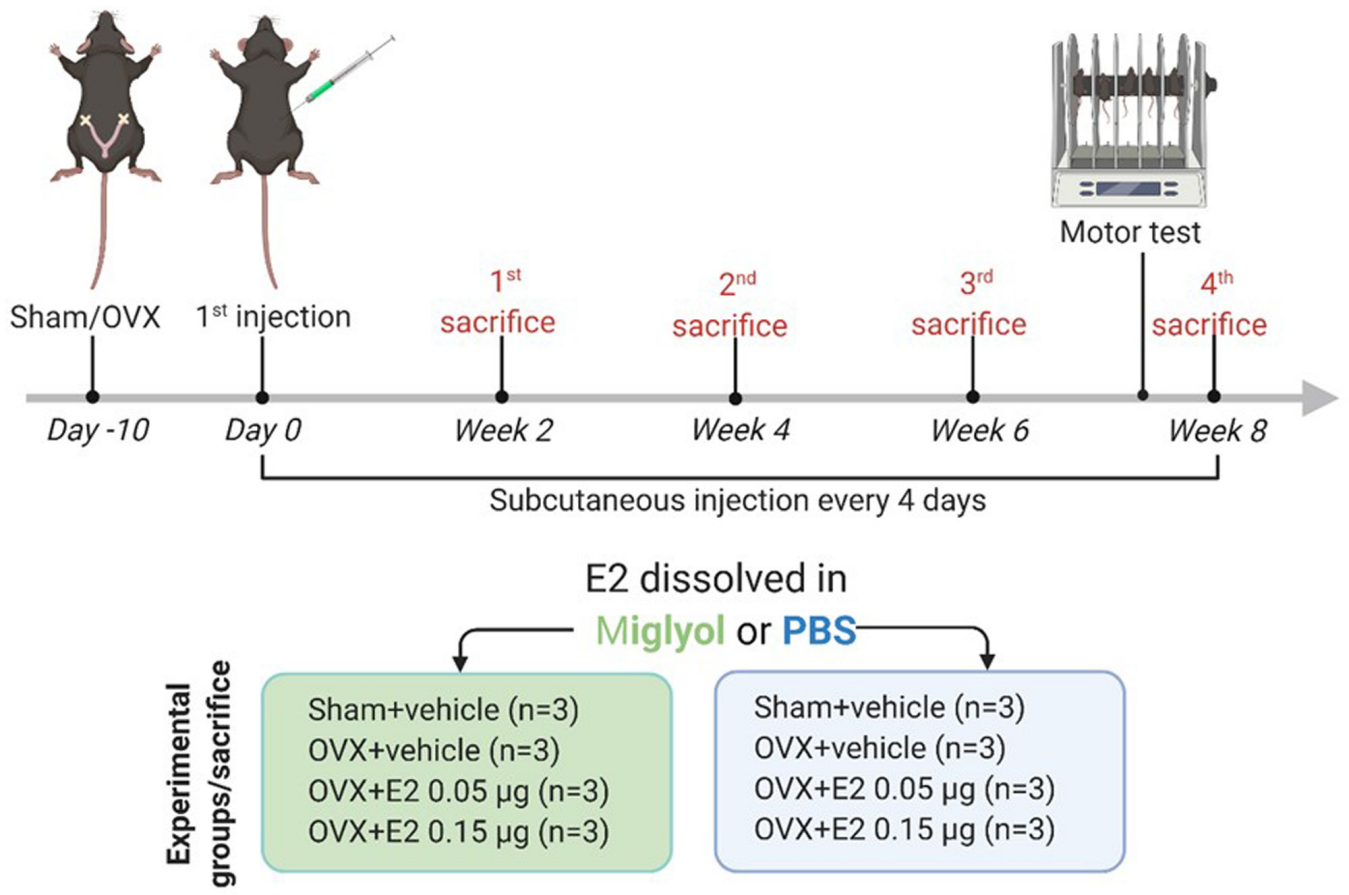
Schematic description of the experimental plan. This figure describes the timeline of the experiments and the experimental groups included in the study.

C57BL/6J mice (Taconic, Denmark) were kept in the animal facility at the University of Gothenburg (Sweden) under regular lighting conditions (12 h light/dark cycles), fed with soya-free laboratory chow and tap water *ad libitum*. Mice were acclimatized for 7 days before initiating the surgical procedures. The experiments were carried out as described in Figure 1. All the experimental procedures were performed in accordance with the ethical permit approved by the Regional Ethical Review Board in Gothenburg, Sweden (Dnr: 2814/2020), which included also the criteria for the earlier termination of the experiment (weight loss and signs of pain and distress), and according to the Institutional Animal Care and Usage Committee ( ARRIVE guidelines ^21^). The sample size (n=3group) was decided to minimize the number of animals and allow the statistical analysis. Animals were randomly allocated in the different experimental groups and randomly sacrificed to minimize potential confounders.

### Ovariectomy

In this study, 96 female mice, 8 weeks old, were employed and underwent OVX or sham surgery. Mice were anesthetized by a mix of isoflurane and oxygen (1.5-2.5 %). A 10 mm incision was made through the shaved and disinfected skin at the lower back area. Then, a 5 mm incision was done in the peritoneum on one side to carefully pull out the ovarian fat pad and excise the ovary by cauterization. The incision in the peritoneum was closed using absorbable sutures. The procedure was repeated on the other side with the second ovary. The external wound on the skin was closed by surgical clips. In mice undergoing sham surgery, the same procedure was performed, but without excising the ovaries. Meloxicam (5mg/kg) was used as a postoperative analgesic.

### Preparation of solutions and estradiol treatments

The oil-based stock solution of E2 (17β-estradiol-3-benzoate, SIGMA Aldrich) (1mg/ml) was prepared by mixing E2 with inert miglyol oil (Miglyol812 OmyaPeralta GmbH, Hamburg, Germany). E2 was dissolved by stirring the solution for 3 hours at 150°C and then further diluted with miglyol to 0.5 µg/ml and 1.5 µg/ml concentrations. 

For the PBS-based formulation, a stock solution (1mg/ml) of E2 dissolved in absolute ethanol was prepared and stored at -20°C. At the time of injections, the stock solution was diluted in PBS to 0.5 µg/ml and 1.5 µg/ml final concentrations.

Mice were allowed to recover from the OVX procedure for 10 days before the initiation of treatments. Sham and OVX mice were divided into different treatment groups receiving subcutaneous (s.c.) injections (100 µl) every four days of E2 (0.05 μg or 0.15 μg/mouse/injection) dissolved in miglyol or PBS, or vehicle (miglyol or PBS). Mice were sacrificed at 2, 4, 6 or 8 weeks after the start of treatments.

### Termination of experiments and tissue collection

Throughout the experiment, all the efforts were made to minimize the pain and distress of the experimental animals. For this purpose, mice were anesthetized with a mixture of ketamine / dexmedetomidine hydrochloride before the procedure. Their body composition was determined using dual-energy x-ray absorptiometry (DXA) scan (UltraFocus ^DXA^, Faxitron Bioptics, Tuscon, AZ). Anesthetized mice were euthanized by exsanguination followed by cervical dislocation. Blood samples were collected for serum isolation. Uterus, thymus, spleen, and liver were dissected, and weights were noted. Femurs were collected for micro-computed tomography (µCT) analysis.

### Micro-computed tomography

After sacrifice, the left hindleg was excised and the femur dissected. The soft tissue was carefully removed from the bone. Samples were fixed in PFA 4% for 3 days and then stored in 70% ethanol. The area for the trabecular bone evaluation was selected starting at 500 µm from the growth plate and extending a further longitudinal distance of 192 µm in the proximal direction. Cortical measurements were performed in the diaphyseal region of the femur starting at 5195 µm from the growth plate and extending a further longitudinal distance of 192 μm in the proximal direction. The selected area was evaluated in a scanning tube providing a voxel size of 4.49 µm isotropically and scanned at 50 kV, 200 µA (Skyscan 1172 scanner; Bruker MicroCT, Aartselaar, Belgium). Samples were kept in paper soaked in PBS to avoid dehydration. Analysis of the morphology and measurement of bone features by μCT was performed using the software CtAN ( 1.13.2.1, Bruker microCT, RRID: SCR_021338).

### Motor tests

To assess the effect of E2 replacement on locomotor ability, two motor tests were performed 2 days before termination of the 8 weeks of treatment experiment. Mice were acclimatized in the procedure room for 1 hour before the start of the tests.

Spontaneous locomotor activity was analyzed using the open-field test. Each mouse was placed into the center of a 60 × 60 × 60 cm chamber to allow free exploration. The experiments were performed for 15 min. The motor parameters were measured by computerized analysis at 3 min intervals. Mice behavior was recorded, and videos were analyzed by using Viewer software ( RRID:SCR_014337; Biobserve, Germany).

Forced locomotor activity was tested by using the rotarod test. Mice were placed on a rotarod apparatus (Panlab, Harvard Apparatus, Spain) and tested for 5 min with constantly increasing acceleration from 4 to 40 r.p.m. The latency to fall was registered for each animal. To exclude differences in learning skills between the groups of mice, each group was assessed over three trials per day for 2 consecutive days. Mice were given a 30 min inter-trial rest interval.

### Sex steroid measurements in serum

Peripheral blood from all mice was collected at termination in 500 µl tubes containing serum gel with clotting activator (Microvette 500 Z-Gel, Sarstedt). The serum was extracted and stored at -80° C until use. Steroids were extracted from serum (200 µl) and concentrations of estradiol, estrone, progesterone, 17β-hydroxiprogesterone, testosterone, dihydrotestosterone (DHT) and androstenedione were analyzed by Liquid Chromatography-Mass Spectrometry (See underlying data, ^22^).

### Data analysis

Data on levels of sex steroids in serum are expressed as median (range) with non-detectable values represented as half of the lowest level of quantification (LLOQ). Statistical analysis was not performed on serum steroid levels due to the presence of values under the detection limit for some hormones, in combination with the low number of samples per experimental group. For all other data Gaussian distribution was assumed and results are expressed as mean ± SEMGraphPad Prism software ( RRID:SCR_002798; GraphPad, San Diego, CA). No animals were excluded from the data analysis. Among the authors of the manuscript, CC was aware of the group allocation at the different stages of the experiment.

## Results

### Effects of OVX and pulsed E2 treatment on serum levels of sex steroids

Mice were subjected to sham or OVX surgery and treated with s.c. injections every 4 days with vehicle (miglyol or PBS) or two different doses of E2 in the corresponding vehicle ( Figure 1). The concentrations of estradiol, estrone, progesterone, 17α-hydroxiprogesterone, testosterone, dihydrotestosterone (DHT), and androstenedione were measured in serum from mice terminated at 8 weeks after start of the treatments.

**Figure 2.**
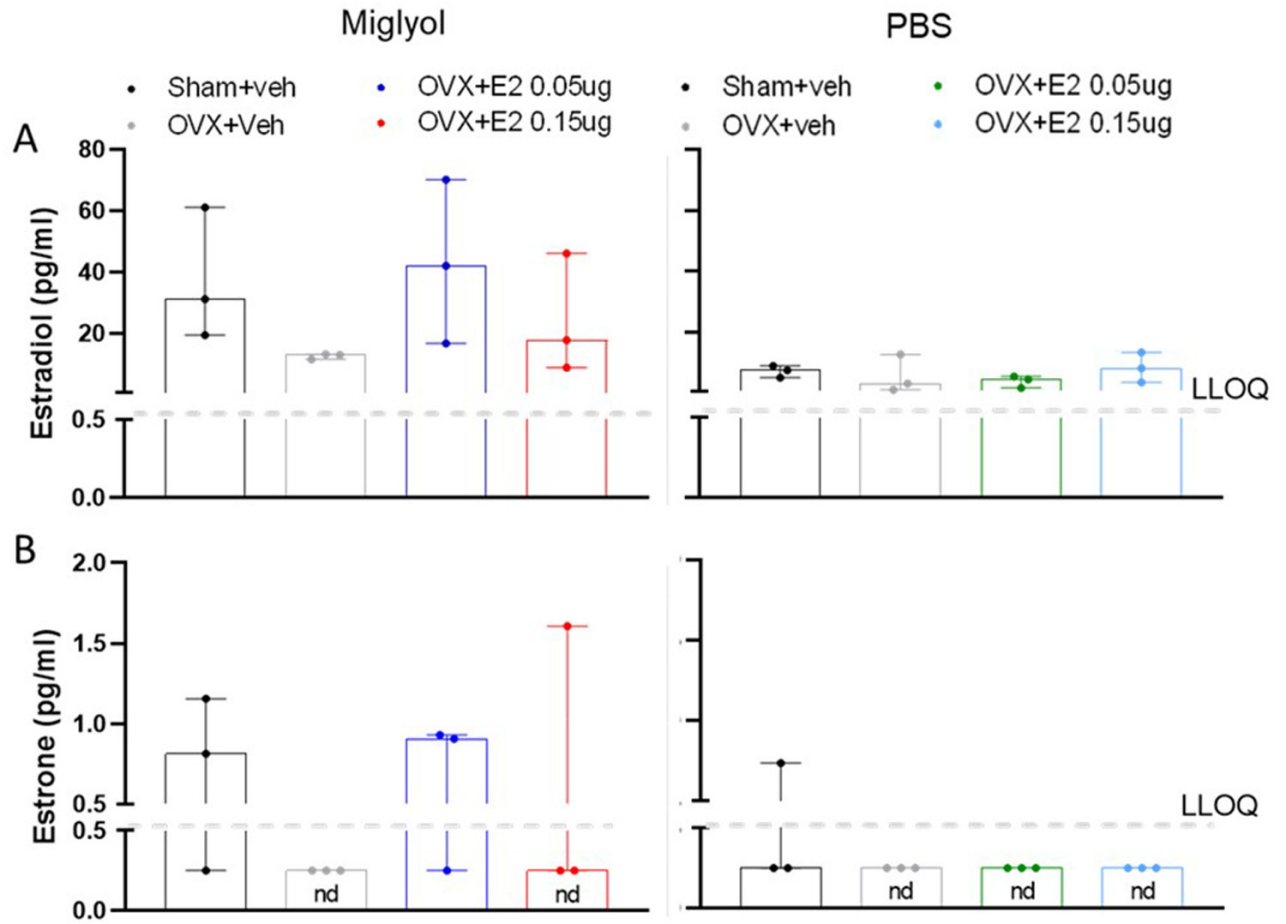
Levels of estradiol and estrone in serum after 8 weeks of treatments. The amount of estradiol ( **A**) and estrone ( **B**) was measured in Sham and OVX mice after 8 weeks of s.c. injections every 4 days with vehicle (miglyol or PBS) or 17 β-estradiol (E2; 0.05 µg/mouse or 0.15 µg/mouse). Data are expressed as median (range); n=3 for each experimental group. (nd = not detectable; LLOQ=lower level of quantification).

***Estradiol and estrone.*** After 8 weeks of treatment, a reduced concentration of estradiol was shown in the miglyol OVX+veh group compared with miglyol sham+veh mice, and both doses of E2 dissolved in miglyol increased the serum level of estradiol compared to OVX+veh. However, in mice receiving the PBS formulation no differences between OVX+veh and any of the other groups could be detected. A comparison between the miglyol sham+veh and PBS sham+veh groups showed that the PBS treated sham group had a tendency towards lower serum concentration of estradiol ( Figure 2A).

The fluctuations of estrone levels in the miglyol preparation groups, showed a similar pattern to that described for estradiol. Estrone levels were generally low, and under the detection limit in the miglyol OVX+veh group. Treatment with both doses of E2 dissolved in miglyol increased the amount of serum estrone compared to OVX mice. However, animals injected with the PBS preparation of E2 displayed estrone values below the detection limit ( Figure 2B).

**Figure 3.**
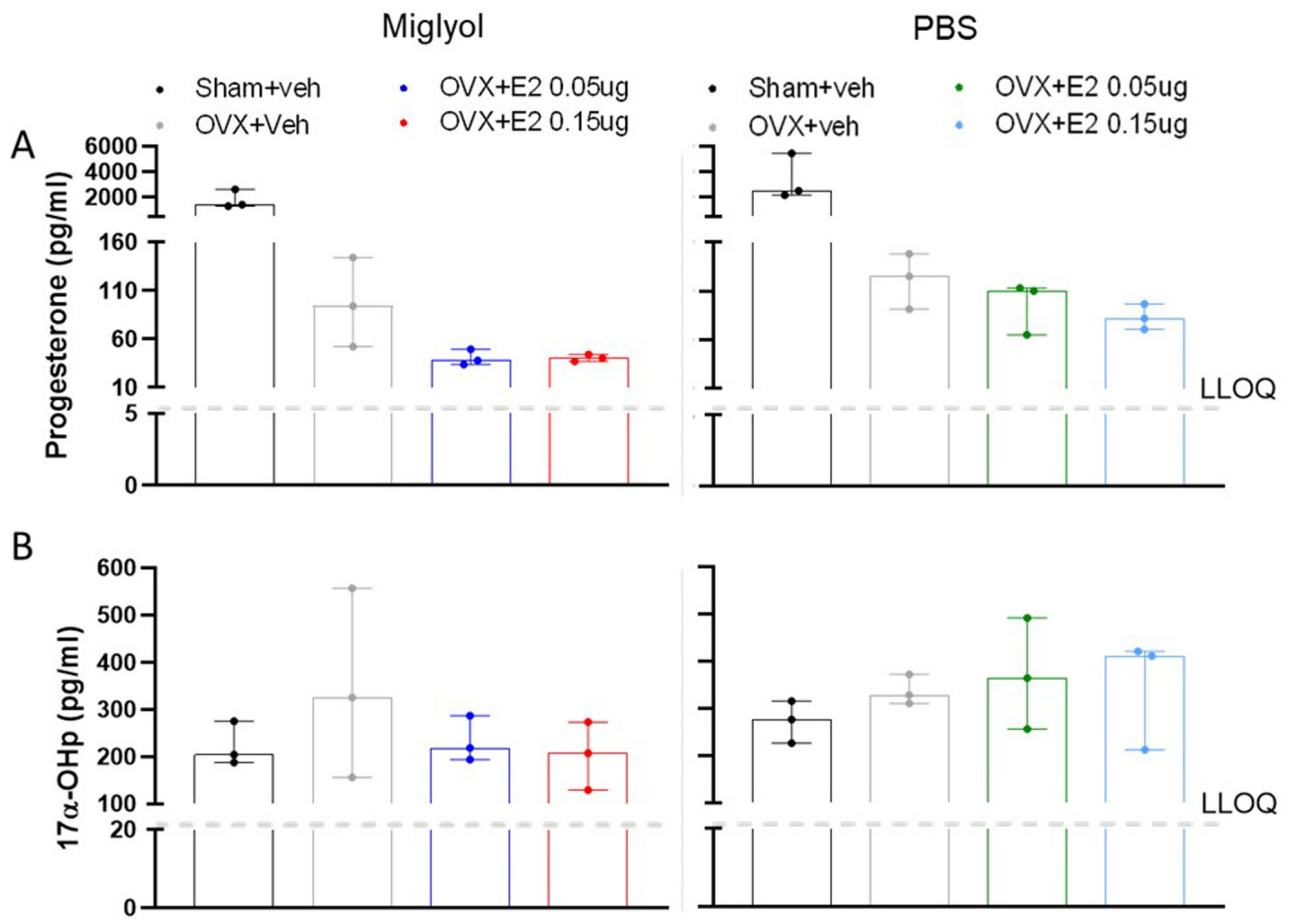
Levels of progesterone and 17α-hydroxyprogesterone in serum after 8 weeks of treatments. The amount of progesterone ( **A**) and 17α-hydroxyprogesterone ( **B**) was measured in Sham and OVX mice after 8 weeks of s.c. injections every 4 days with vehicle (miglyol or PBS) or 17 β-estradiol (E2; 0.05 µg/mouse or 0.15μg/mouse). Data are expressed as median (range); n=3 for each experimental group. (LLOQ = lower level of quantification).

***Progesterone and 17β-hydroxiprogesterone.*** As expected, OVX induced a reduction in serum progesterone levels. Treatments with E2, dissolved in miglyol or PBS, did not affect the serum concentrations of progesterone ( Figure 3A).

Serum levels of 17β-hydroxiprogesterone were unaltered between the groups after 8 weeks of treatment ( Figure 3B).

**Figure 4.**
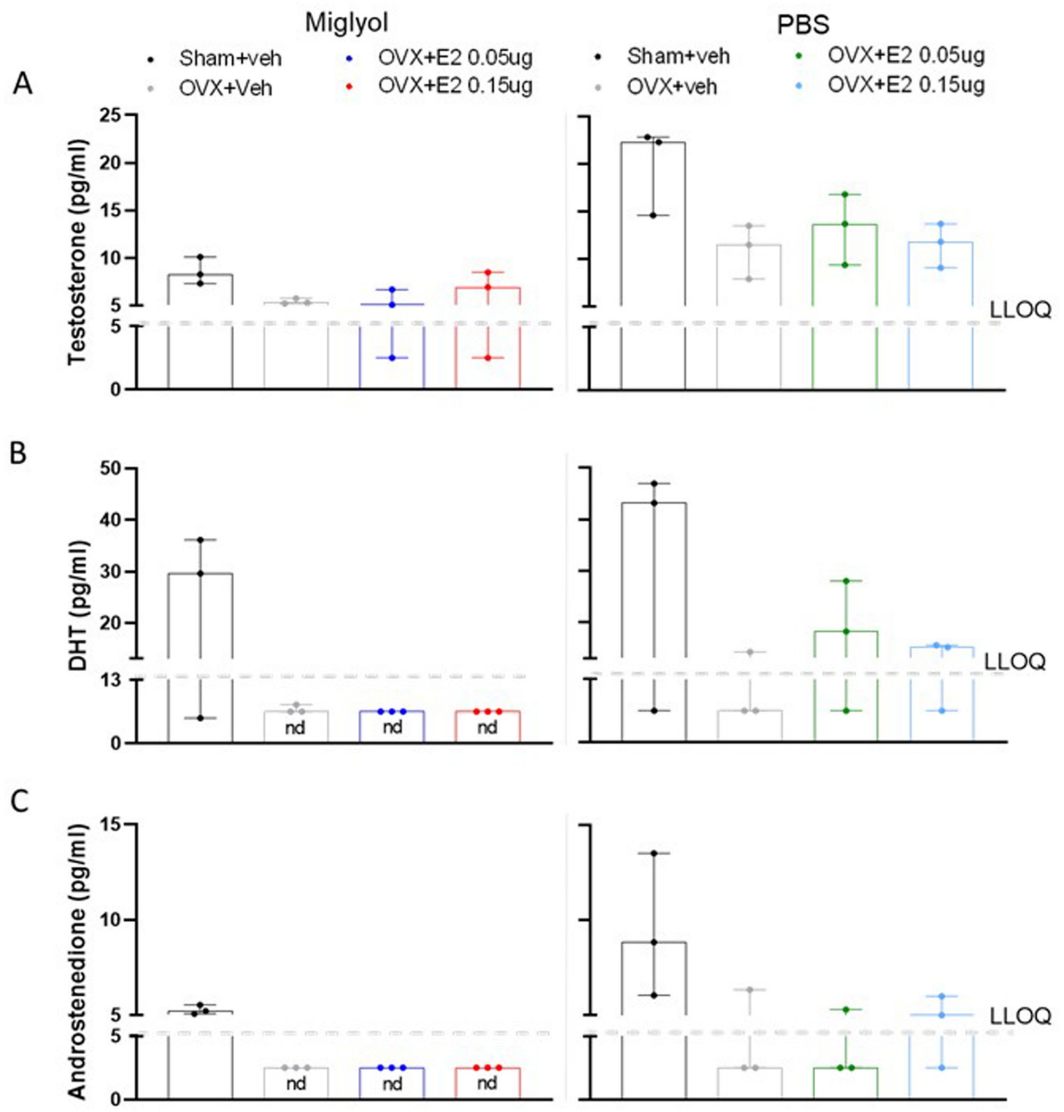
Levels of testosterone, dihydrotestosterone (DHT) and androstenedione in serum after 8 weeks of treatments. The amount of testosterone ( **A**), dihydrotestosterone (DHT; **B**) and androstenedione ( **C**) was measured in Sham and OVX mice after 8 weeks of s.c. injections every 4 days with vehicle (miglyol or PBS) or 17 β-estradiol (E2; 0.05 µg/mouse or 0.15 µg/mouse). Data are expressed as median (range); n=3 for each experimental group. (nd = not detectable; LLOQ=lower level of quantification).

***Testosterone, dihydrotestosterone (DHT) and androstenedione.*** The levels of testosterone tended to decrease in OVX+veh groups for both formulations compared to the sham+veh groups in mice terminated after 8 weeks. Testosterone concentrations were not altered by E2 replacement, in neither the miglyol nor the PBS preparation groups ( Figure 4A).

Serum levels of DHT were reduced after OVX in both miglyol and PBS preparations. Treatment with E2 dissolved in miglyol resulted in DHT levels under the detection limit after 8 weeks, while E2 dissolved in PBS partially restored the serum levels of DHT ( Figure 4B).

The serum levels of androstenedione in the different groups resembled those for DHT ( Figure 4C). Androstenedione was reduced in the OVX groups after 8 weeks of treatment compared to the sham mice, with values under the detection limit in the miglyol OVX+veh and OVX+E2 groups.

### Effects of OVX and pulsed E2 treatment over time in soft tissues

**Figure 5.**
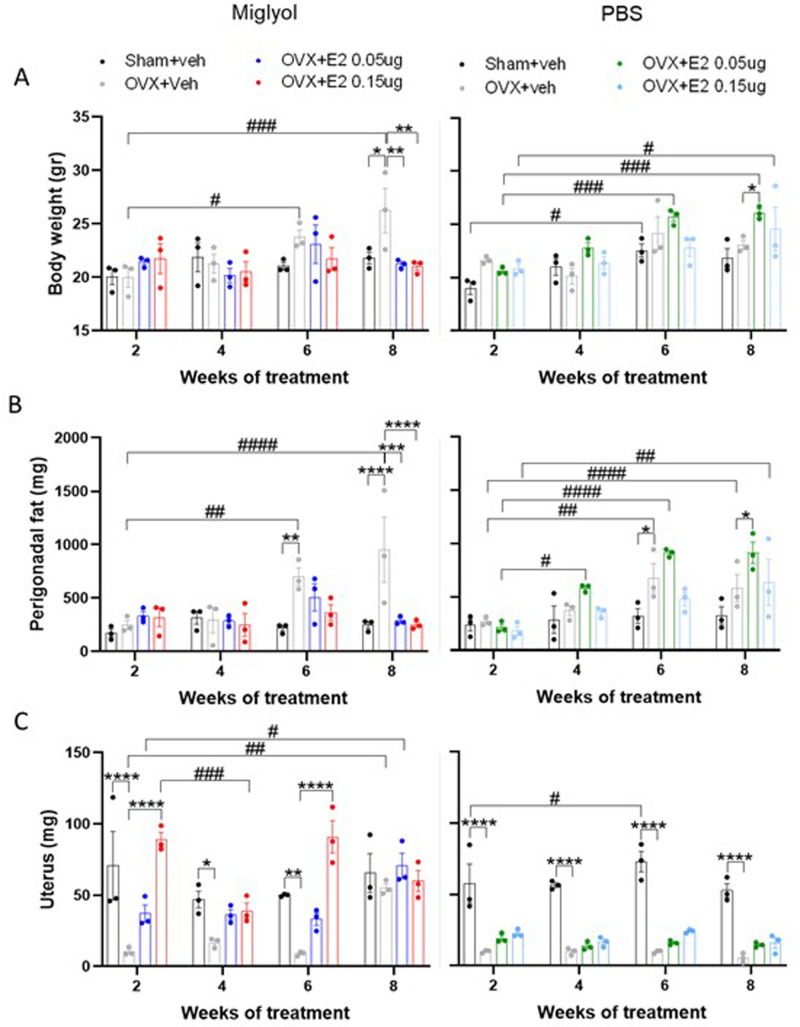
Body weights and weights of soft tissues. Mice were subjected to Sham or OVX surgery and treated with s.c. injections of vehicle (miglyol or PBS) or 17β-estradiol (E2; 0.05µg/mouse or 0.15µg/mouse) every 4 days. Experiments were terminated at 2, 4, 6 and 8 weeks after the start of treatments. Body weight ( **A**), perigonadal fat ( **B**) and uterus ( **C**) weights were determined at sacrifice. Data are expressed as mean±SEM; n=3 for each experimental group. * statistical differences between sham+veh vs OVX+veh; or OVX+veh vs the E2 treatment groups; # statistical difference vs week 2 of the same treatment group. *or #, p<0.05; **or ##, p<0.01; ***or ###, p<0.001; ***** or ####, p<0.0001; Two-way ANOVA followed by Dunnet’s post-hoc test).

The body weight and the weight of perigonadal fat, uterus, thymus, liver, and spleen were measured at the time of each termination ( Figure 5; extended data figure. 1 ^21^). The miglyol sham+veh mice had constant body weight for the whole duration of the experiment. However, the miglyol OVX+veh group showed a significant increase in body weight compared to the sham+veh group after 8 weeks of treatment, which was efficiently prevented by treatment with both doses of E2 dissolved in miglyol ( Figure 5A). On the contrary, the PBS OVX+veh group did not differ in body weight from PBS sham+veh mice at any time-point. Treatment with E2 in PBS resulted in a gradual increase of the body weight compared to body weights of the PBS OVX+E2 groups measured after 2 weeks of treatment and reached a significant increase at 6 and 8 weeks of treatment for the low and high dose of E2, respectively ( Figure 5A).

Consistent with the results of body weights, the perigonadal fat in the miglyol OVX+veh group increased significantly at 6 and 8 weeks of treatment compared to the miglyol sham+veh group ( Figure 5B). Furthermore, miglyol E2 treatment of OVX mice prevented perigonadal fat accumulation. At 6 weeks of treatment, the PBS OVX+veh group had significantly increased perigonadal fat weight compared to the PBS sham+veh group. Consistent with the results of body weights there was a gradual increase in perigonodal fat in mice treated with both doses of PBS E2. After 8 weeks of treatment, the perigonadal fat of the PBS OVX+E2 0.05µg group was also significantly higher compared to the PBS OVX+veh mice ( Figure 5B).

As expected, the uterus weight was substantially lower in the OVX+veh mice compared to sham+veh mice in both miglyol and PBS groups ( Figure 5C). An increase of the uterus weight was induced in the miglyol OVX+E2 0.15 µg group, starting at 2 weeks of the treatment. At the termination after 8 weeks of treatment, no differences between the miglyol OVX+E2 groups compared to the OVX+veh group were detected due to the unexpectedly increased uterus weight in miglyol OVX+veh mice ( Figure 5C, left). On the contrary, injections of E2 dissolved in PBS did not increase the uterus weight when compared to the PBS OVX+veh mice ( Figure 5C, right).

After 2 weeks of treatment, the PBS OVX+veh group had increased thymus weight compared to the PBS sham+veh mice, however the differences were equalized during the later time points. No major differences between the groups were detected for liver and spleen weights in either miglyol or PBS preparations (Extended data, figure. 1A–C, ^21^).

### Analysis of the body composition after OVX and pulsed E2 treatment

**Figure 6.**
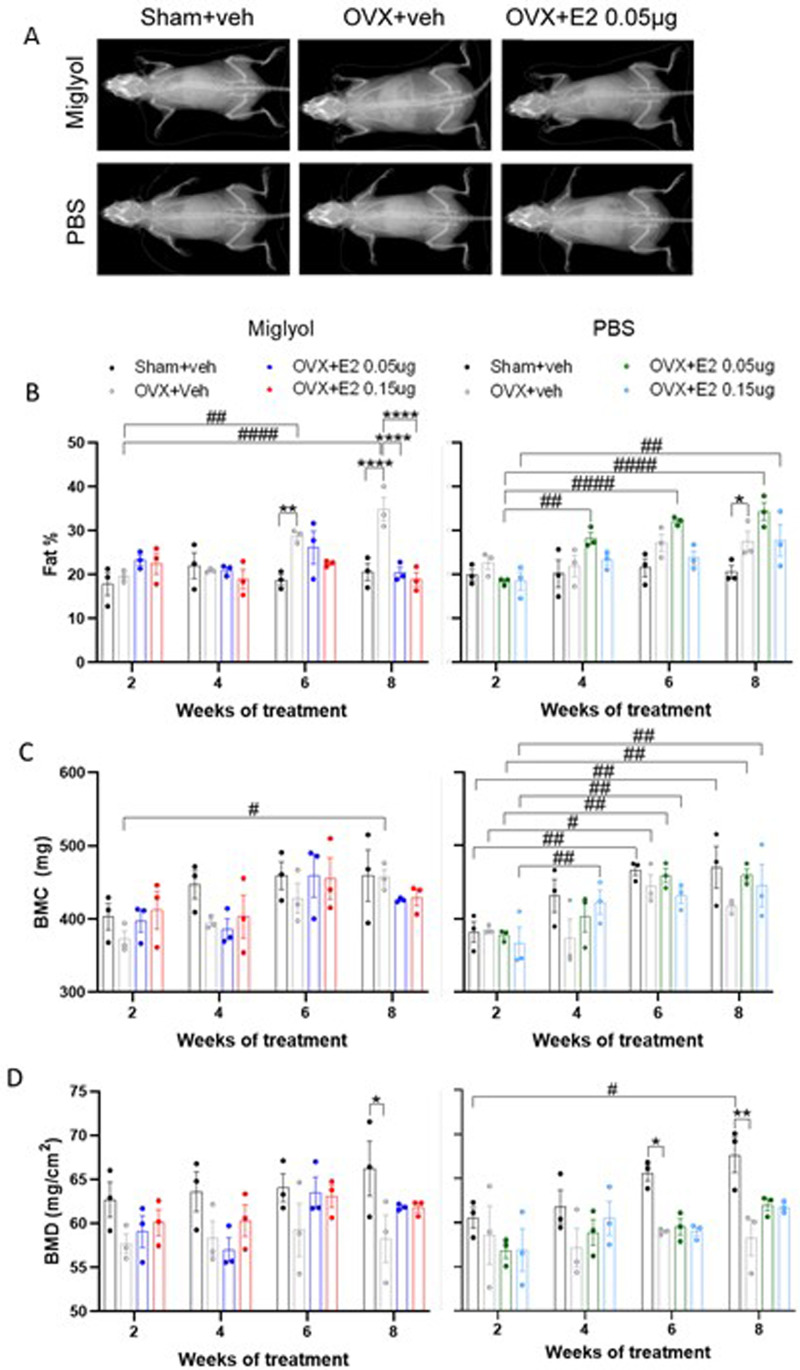
Analysis of body composition by DXA. Mice were subjected to Sham or OVX surgery and treated with s.c. injections of vehicle (miglyol or PBS) or 17β-estradiol (E2; 0.05 µg/mouse or 0.15 µg/mouse) every 4 days. The analyses were performed using a dual energy x-ray absorptiometry (DXA) scanner before sacrifice after 2, 4, 6 and 8 weeks of treatment. Representative DXA scan images for the experimental groups after 8 weeks of treatment ( **A**). Percentage of fat ( **B**), bone mineral content (BMC; **C**) and bone mineral density (BMD; **D**) were determined for the whole body. Data are expressed as mean±SEM; n=3 for each experimental group. * statistical differences between sham+veh vs OVX+veh; or OVX+veh vs the E2 treatment groups; # statistical difference vs week 2 of the same treatment group. *or #, p<0.05; **or ##, p<0.01; ***or ###, p<0.001; ***** or ####, p7lt;0.0001; Two-way ANOVA followed by Dunnet’s post-hoc test).

Before each sacrifice, the whole-body bone, fat and lean mass composition were measured using DXA scan ( Figure 6A) ( Underlying data ^22^). The percentage of fat in miglyol OVX+veh mice increased over time and reached statistically significant differences at weeks 6 and 8 compared to 2 weeks of treatment. After 8 weeks of treatment, both groups of miglyol OVX+E2 displayed decreased levels of body fat compared to the miglyol OVX+veh group ( Figure 6B, left). On the contrary, both the low and the high dose of E2 dissolved in PBS induced an increase in whole body fat over time, compared to the same treatment at week 2 ( Figure 6B, right). No differences between treatments were detected in lean mass ( Extended data, figure. 2A, ^21^).

In the miglyol groups, the OVX+veh mice showed an increase of bone mineral content (BMC) and bone area after 8 weeks of treatment compared to week 2 ( Figure 6C, left; extended data, figure. 2B, left, ^21^). At week 4, the BMC of PBS OVX+E2 0.15µg mice were higher compared to week 2 of treatment. The other PBS experimental groups also showed an increase of the BMC and bone area at weeks 6 and 8 compared to the same treatment at week 2 ( Figure 6C, right, extended data 2B, right, ^21^).

The bone mineral density (BMD) was significantly lower in miglyol OVX+veh mice compared to the sham+veh group at 8 weeks of treatment. Neither the high nor the low dose of miglyol E2 treatments affected the BMD compared to OVX+veh ( Figure 6D, left). Similarly, a decrease in BMD was detected in the PBS OVX+veh group at weeks 6 and 8 compared to the PBS Sham+veh groups ( Figure 6D, right).

### Effects of OVX and pulsed E2 replacement on the features of long bones

**Figure 7.**
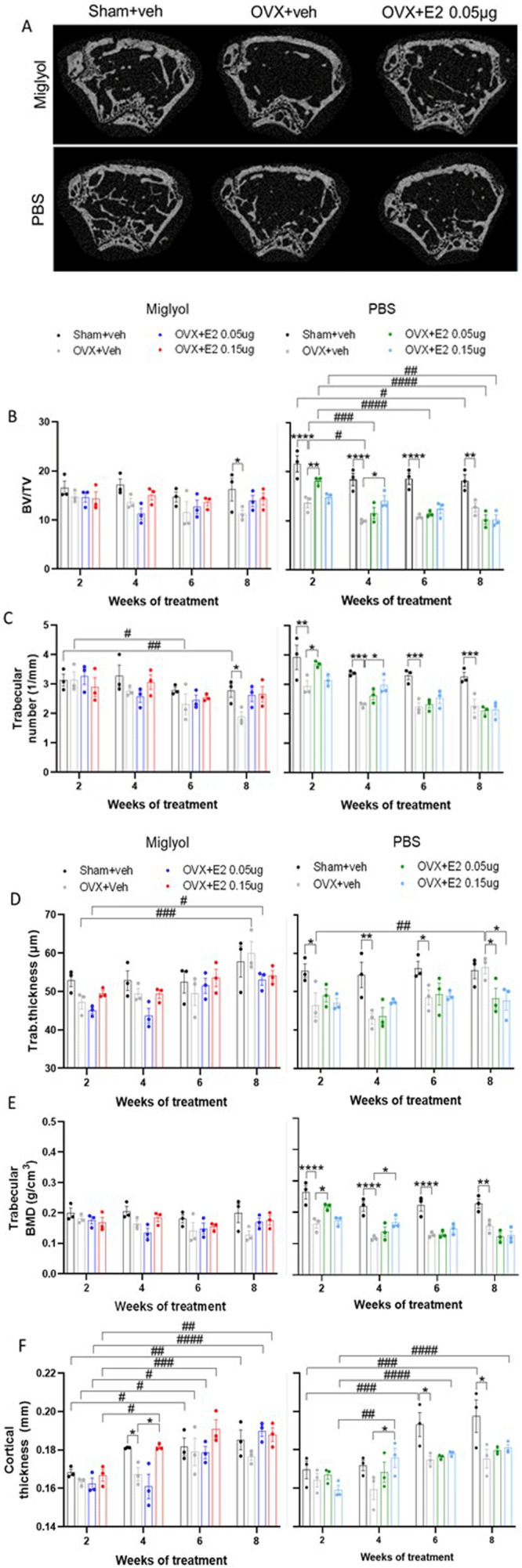
Features of femur analysed by µCT analysis. Mice were subjected to Sham or OVX surgery and treated with s.c. injections of vehicle (miglyol or PBS) or 17β-estradiol (E2; 0.05µg/mouse or 0.15µg/mouse) every 4 days. Experiments were terminated at 2, 4, 6 and 8 weeks after the start of treatments and femurs were dissected. Trabecular and cortical parameters of the femoral bone were determined by micro-computed tomography (µCT) analysis. Representative µCT scan pictures of the trabecular and cortical femur for the experimental groups after eight weeks of treatment ( **A**). µCT analyses of bone volume/total volume (BV/TV); **B**), trabecular number ( **C**), trabecular thickness ( **D**), trabecular bone mineral density (BMD; **E**), and cortical thickness ( **F**) were performed on femur. Data are expressed as mean±SEM; n=3 for each experimental group. * statistical differences between sham+veh vs OVX+veh; or OVX+veh vs the E2 treatment groups; # statistical difference vs week 2 of the same treatment group. *or #, p<0.05; ##, p<0.01; **** or ####, p<0.0001; Two-way ANOVA followed by Dunnet’s post-hoc test).

Micro-computed tomography was performed on dissected femurs of all mice for each time point ( Figure 7A) ( Underlying data ^22^). After 8 weeks of treatment a decrease in bone volume over total volume (BV/TV) was detected in both miglyol and PBS OVX+veh mice compared to the sham counterparts ( Figure 7B). The loss of bone was faster (2 weeks) in OVX mice receiving PBS-vehicle. No significantly protective effects on BV/TV were detected by E2 replacement in the miglyol groups ( Figure 7B, left). An early protective effect (2 weeks) was measured in the PBS OVX+E2 mice ( Figure 7B, right).

After 8 weeks of treatment with miglyol OVX+veh, mice displayed reduced trabecular number compared to sham+veh. Treatment with both doses miglyol E2 resulted in a trend towards increased trabecular number ( Figure 7C, left). In the PBS OVX+veh group, the trabecular number was significantly reduced at all time points compared to PBS sham+veh ( Figure 7C, right).

For trabecular thickness similar patterns were detected between the miglyol and PBS OVX+veh groups at week 8, showing increased trabecular thickness compared to the corresponding OVX+veh groups at week 2 ( Figure 7D).

The trabecular BMD was reduced in the PBS OVX+veh mice compared to the PBS sham+veh group, constantly over time ( Figure 7E, right). No significant differences were detected for any of the miglyol experimental groups ( Figure 7E, left).

Loss of cortical thickness was detected in the miglyol OVX+veh group after 4 weeks of treatment, and the high dose of E2 (0.15µg) in miglyol protected from the decrease in cortical thickness ( Figure 7F, left). In addition, both sham+veh, OVX+veh, and treatment of OVX mice with both doses of miglyol E2, resulted in an increase in cortical thickness over time ( Figure 7F, left). For the PBS group the cortical thickness was significantly decreased in OVX+veh compared to sham+veh starting at week 6 and the high dose E2 treatment increased the cortical thickness from 4 weeks of treatment ( Figure 7F, right).

The cortical area increased over time compared to week 2 in all the experimental groups of mice receiving miglyol, as well as in the PBS sham+veh group from 6 weeks of treatment ( Extended data, figure. 3A, ^21^).

The porosity of the bone was decreased in the PBS OVX+veh mice compared to the PBS sham+veh group at 4 weeks after the start of the treatments. Also, an overall reduction over time among the PBS OVX treatment groups were detected starting at week 6 ( Extended data, figure. 3B, ^21^).

### Effects of OVX and pulsed E2 replacement on motor ability

**Figure 8.**
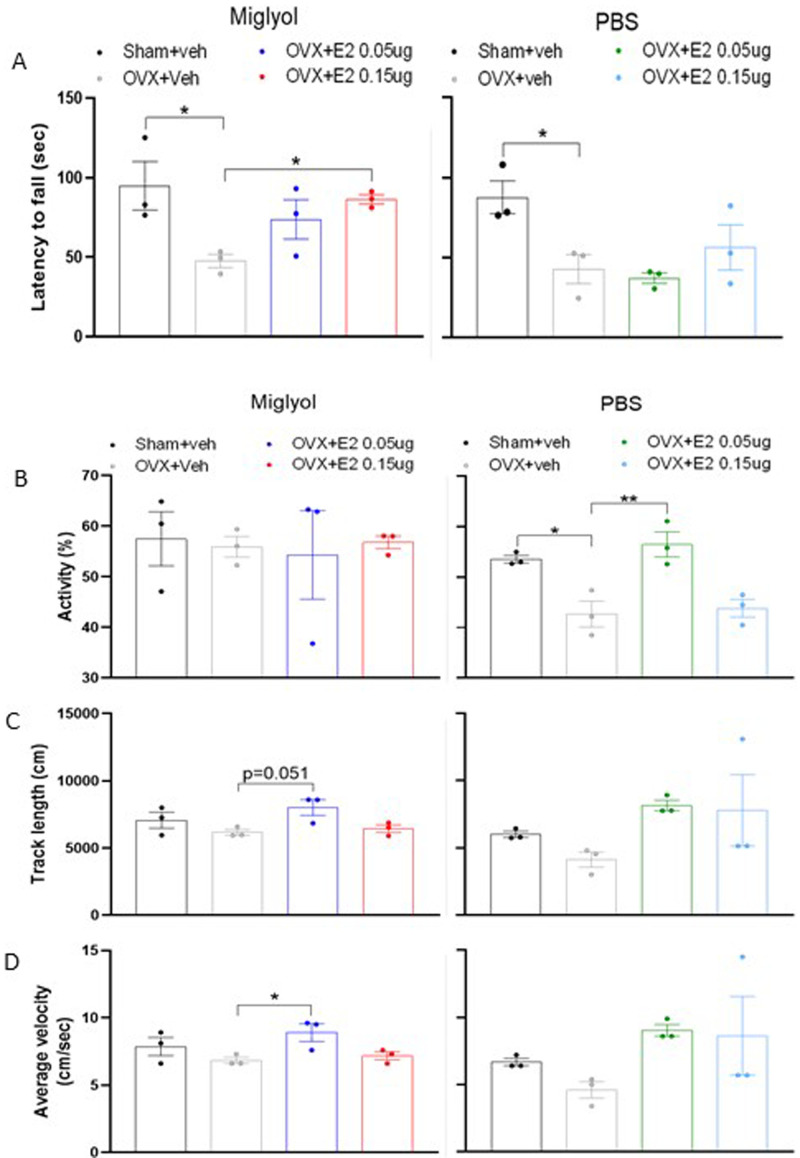
Motor ability determined by rotarod and open field tests after 8 weeks of treatments. Mice were subjected to Sham or OVX surgery and treated with s.c. injections of vehicle (miglyol or PBS) or 17β-estradiol (E2; 0.05 µg/mouse or 0.15 µg/mouse) every 4 days. After 8 weeks of treatments, motor behavior was tested using the rotarod test for measurement of latency to fall ( **A**) and the open field test to measure activity ( **B**), track length ( **C**) and average velocity ( **D**). Data are expressed as mean±SEM; n=3 for each experimental group. One-way ANOVA followed by Dunnet’s post-hoc test was used to analyze differences between sham+veh vs OVX+veh; or OVX+veh vs the E2 treatment groups. *, p<0.05; **, p<0.01.

The capacity to run on the rotarod (set to a constant acceleration) was reduced in OVX+veh mice compared to the sham+veh in both PBS and miglyol groups ( Figure 8A). An improvement in the latency to fall was registered in OVX mice treated with miglyol E2 0.15µg compared to the OVX+veh mice ( Figure 8A, left).

In the open field test, miglyol OVX+veh mice tended to move less and slower compared to the sham+veh operated mice ( Figure 8B–D, left). A similar and more pronounced pattern was registered in the mice from the PBS groups. In this group, E2 replacement with the lower dose was able to restore the activity parameters ( Figure 8B–D, right).

## Discussion

There is a clear female bias in the development of certain pathologies and estrogens are associated with differences in disease progression. However, the specific mechanisms underlying the effects of estrogens, are still unclear. Experimental mouse models constitute a powerful tool that can be used to define the effect of estrogen on specific cells, tissues and organs. The goal of this study was to determine a dose and preparation of 17β-estradiol-3-benzoate (E2), which restores ovariectomy (OVX)-induced effects in mice to the physiological levels of sham operated controls. The results from this study are intended to be used as a guide in the planning of experiments that aims to define the specific effects of treatment with a physiological dose of E2 in experimental mouse models.

Surgical removal of the ovaries was performed in order to mimic a postmenopausal state. OVX mice were treated with subcutaneous E2 injections and monitored at 2 weeks intervals for up to 8 weeks of treatment. The doses of E2 used, 0.05µg or 0.15µg per mouse/per injection, were previously reported as concentrations equivalent to estrus and anestrus levels in mice ^23^. E2 was injected every 4 days to resemble the length of the estrus cycle in mice ^24^. Two vehicles for dissolving E2 were tested: miglyol is described as an inert oil used for slow and sustained absorption of the drug from the oily pocket created after the injection in the subcutaneous area; PBS is used for faster absorption ^25, 26^.

Sex steroid concentrations were analyzed in serum collected at the time of sacrifice. As expected, OVX resulted in reduced levels of estradiol, progesterone, and testosterone, hormones that are primarily produced by the ovaries. The injections of E2 dissolved in oil did not exceed the control (sham) concentrations of serum estradiol after 8 weeks of treatment, indicating that both tested E2 doses were within the physiological range. However, lower levels of estradiol were found in serum when the drug was dissolved in PBS, suggesting a rapid wash out or reduced drug absorption.

Lack of estrogen provoked fat accumulation and a consequent increase of body weight, a phenotype that is linked to increased feed efficiency and reduced lipolysis in white adipose tissue ^27, 28^. Replacement of E2 starting 10 days after OVX prevented fat accumulation. Interestingly the lower dose of estradiol delivered with PBS had the opposite effect and induced an increase in the body weight as a consequence of perigonadal fat and total body fat accumulation. This difference in response to the same dose injected could be due to a lower absorption of the aqueous formulation or to a rapid washout making the E2 less bioavailable compared to the E2 dissolved in miglyol. The biological effects of E2 and the expression of estrogen receptors are strictly associated with its concentration, a pharmacological mechanism known as a non-monotonic dose-response ^29^.

The uterus is a fast-responding organ that is sensible to hormone alterations. The uterus size changes during the menstrual cycle, enlarging and shrinking in response to sex hormone availability, in particular estradiol and progesterone. As expected, the lack of ovaries and the consequent reduction of estradiol and progesterone reduced the uterus weight. The uterus was enlarged to the same levels as the sham controls after treatment with E2 dissolved in oil. Unexpectedly, at 8 weeks after the surgery and treatment with oil as vehicle, the uterus of OVX mice had increased and reached similar size as the sham controls. We speculate that this could be related to local production of estrogens in the perigonadal fat ^30^. A slight increase of the uterus weight was detected in mice treated with E2 dissolved in PBS, an indication that the concentration of E2 delivered with PBS as solvent is much lower compared to E2 dissolved in miglyol. A previous study demonstrated that injections of mice with E2 dissolved in peanut oil (2µg/mouse every 4 days) increased the uterus size up to 4 times the size of the control mice ^31^. For studies of pain and mobility in mice, an important characteristic is keeping the uterus at the same size as the control. An oversized uterus and the hypertrophy of the vaginal tract will induce pain, reduced mobility, and will alter the feeding behavior. Furthermore, an increase of urinary infections caused by a large uterus will impair the general health of the animals and alter the final results of the experiment ^32^. Together, this highlights the importance of choosing a physiological dose of E2 for studies where E2 treatment is performed. Bone parameters are also affected by estrogens to a large extent. E2 controls bone formation and bone resorption by effects on osteoclasts and osteoblasts thereby affecting the amount of bone, its microarchitecture, and its quality in terms of strength and physical properties ^33^. For example, estrogen deficiency results in sub-clinical inflammation with increased expression of pro-inflammatory cytokines including Interleukin-1 (IL-1), IL-6 and Tumor Necrosis Factor (TNF), which leads to an increase in osteoclastogenesis ^34^. Development of osteoporosis was visible already at an early point in the OVX mice, with a reduction in the number and thickness of the femoral trabeculae. At 8 weeks, an increase in BMC, bone area and trabecular thickness was detected, most likely representing a compensatory mechanism with the aim to contrast the reduced bone mineral density and trabecular number and maintain bone strength. A similar compensatory effect was previously described in tibia of OVX rats ^35^.

The fact that some women suffer from depression in the premenstrual, postpartum and perimenopausal periods is well documented ^36, 37^. Estradiol affects the central nervous system by promoting spontaneous physical activity, which supports lipolysis in the white adipose tissue and stimulates the reduction of body mass ^28, 38^. In this study, OVX mice that were tested for spontaneous locomotor activity, moved less and slower compared to the sham-operated control mice at 8 weeks post-surgery. In the rotarod test, OVX mice showed a pain-associated behavior, which was also described at 8 weeks after OVX in a previous study ^39, 40^.

## Conclusion

In this study we found that subcutaneous injections of OVX mice with low dose E2 (either 0.05 or 0.15µg/mouse dissolved in miglyol) every 4 ^th^ day, results in serum levels of estradiol comparable to sham operated control mice. Furthermore, both doses of E2 treatment restore body fat composition, rescue the OVX induced decrease in BMD and increase motor ability to the level of sham operated control mice. Thus, pulsed E2 treatment with low dose E2 dissolved in miglyol mimics the endogenous level of estradiol produced by the ovaries in mice. A milder effect was detected when E2 was dissolved in PBS compared to miglyol, indicating a rapid release and wash out of the hormones from the subcutaneous area, and that E2 dissolved in the aqueous vehicle could be more suitable for short term experiments.

The limitation of this study is the small sample size. Despite this, the study provides a useful tool for the planning of experiments, which aims to investigate the effects of a physiological dose of E2 administered in a pulsed fashion, thereby resembling the cyclic fluctuations of estradiol in normal mice and avoid duplication of effort.

## Data availability

### Underlying data

Zenodo: Pulsed administration for physiological estrogen replacement in mice.

https://doi.org/10.5281/zenodo.5036241 ^22^

The project contains the following underlying data:

- DXA data: Dual energy x-ray absorptiometry (DXA) measurements for the whole body and lumbar spine of each mouse included in the study.
- Motor tests: Video files from the open field test and excel files including raw data from the open field and rotarod test.
- Steroid: Excel files including raw data from the steroids measurement.
- Tissues and body weight: Information about the weight of the body and organs of the animals included in this study. (Description of data).
- uCT: Excel files including the raw data obtained from the micro-computed
- Tomography analysis of the femur collected from each mouse included in this study

### Extended data

Zenodo: Pulsed administration for physiological estrogen replacement in mice.

https://10.5281/zenodo.5140017 ^21^.

The project contains the following extended data:

- Supplemental figures: The file includes extra figures and data from the analysis of the organs weight, DXA, and uCT measurement.

Data are available under the terms of the Creative Commons Zero "No rights reserved" data waiver (CC0 1.0 Public domain dedication).

### Reporting guidelines

Zenodo: ARRIVE checklist for “Pulsed administration for physiological estrogen replacement in mice”. https://10.5281/zenodo.5140017 ^21^.

The file is available under the terms of the Creative Commons Attribution 4.0 International.
