# Supplementary material for: Pulsed administration for physiological estrogen replacement in mice
Source: F1000Res. 2021 Aug 16;10:809. [Version 1] doi: 10.12688/f1000research.54501.1 (PMC8609397; doi:10.12688/f1000research.54501.1)
Supplement: Peer review history

# Peer review: Reviewer response for version 1

**DOI:** 10.5256/f1000research.57994.r98755

**Published:** 2021-11-22

**Authors:** Julia O. Fedotova

**Affiliations:** 1 Laboratory of Neuroendocrinology, I.P. Pavlov Institute of Physiology RASci., St. Petersburg, Russian Federation

This is a very nice study concerning the effects of pulsed subcutaneous injections with 17β-estradiol-3-benzoate (E2) every 4 days in healthy ovariectomized (OVX) mice. The present work creates a useful tool for the planning of experiments, which aims to investigate the effects of a physiological dose of E2 administered in a pulsed way.

 I recommend to continue this study with OVX animals of different age using a physiological dose of E2 administered in a pulsed regime.

Is the rationale for developing the new method (or application) clearly explained?

Yes

Is the description of the method technically sound?

Yes

Are the conclusions about the method and its performance adequately supported by the findings presented in the article?

Yes

If any results are presented, are all the source data underlying the results available to ensure full reproducibility?

Yes

Are sufficient details provided to allow replication of the method development and its use by others?

Yes

Reviewer Expertise:

neuropsychopharmacology, psychoneuroendocrinology, hormones and behavior, affective-related disorders, cognitive impairments

I confirm that I have read this submission and believe that I have an appropriate level of expertise to confirm that it is of an acceptable scientific standard.

# Peer review: Reviewer response for version 1

**DOI:** 10.5256/f1000research.57994.r92272

**Published:** 2021-08-31

**Authors:** Narendra Verma

**Affiliations:** 1 Division of Endocrinology, Diabetes and Metabolism, Department of Medicine, New York University, New York, NY, USA

In this nice study, the authors show that distribution of 17β-estradiol-3-benzoate (E2) efficacy depends on the solvent and dose frequency. The authors did compare two solvents miglyol and PBS and showed their profound effects, however, I do have some minor concerns:

1. To add some more literature about the estrogens and their role in bone development.
2. There are some typo errors the authors should fix 

Is the rationale for developing the new method (or application) clearly explained?

Yes

Is the description of the method technically sound?

Yes

Are the conclusions about the method and its performance adequately supported by the findings presented in the article?

Yes

If any results are presented, are all the source data underlying the results available to ensure full reproducibility?

Yes

Are sufficient details provided to allow replication of the method development and its use by others?

Yes

Reviewer Expertise:

Bone disorder, Obesity and diabetes

I confirm that I have read this submission and believe that I have an appropriate level of expertise to confirm that it is of an acceptable scientific standard.
